# Obstructive Sleep Apnea: The Effect of Bariatric Surgery After Five Years—A Prospective Multicenter Trial

**DOI:** 10.1007/s11695-024-07124-5

**Published:** 2024-03-08

**Authors:** Pipsa Peromaa-Haavisto, Markku Luostarinen, Risto Juusela, Henri Tuomilehto, Jyrki Kössi

**Affiliations:** 1 Department of Surgery, Tampere University Hospital Hatanpää, PL2000, FIN-33521 Tampere, Finland; 2 Päijät-Häme Central Hospital, 15850 Lahti, Finland; 3 Aava Medical Center, 00100 Helsinki, Finland; 4 Vaasa Central Hospital, 65130 Vaasa, Finland

**Keywords:** Bariatric surgery, Obstructive sleep apnea, Effect of bariatric surgery, Obesity surgery, Metabolic surgery, OSA

## Abstract

**Background:**

The prevalence of obstructive sleep apnea (OSA) is high among the bariatric surgery candidates. Obesity is the most important individual risk factor for OSA. The aim of this study was to investigate the effect of a laparoscopic Roux-en-Y gastric bypass (LRYGB) on OSA 5 years after the surgery.

**Patients and Methods:**

In this prospective multicenter study, standard overnight cardiorespiratory recording was conducted to 150 patients at baseline prior to bariatric surgery. A total of 111 (73.3%) patients of those had OSA. Cardiorespiratory recordings at 5 years after surgery were available for 70 OSA patients. The changes in anthropometric and demographic measurements including age, weight, body mass index (BMI), and waist and neck circumference were evaluated. Also, a quality of life (QoL) questionnaire 15D administered in a baseline was controlled at 5-year follow-up visit.

**Results:**

At 5-year OSA was cured in 55% of patients, but moderate or severe OSA still persisted in 20% of patients after operation. Mean total AHI decreased from 27.8 events/h to 8.8 events/h (*p* < 0.001) at 5-year follow-up. A clinically significant difference in QoL was seen in mobility, breathing, sleeping, usual activities, discomfort and symptoms, vitality and sexual activity. The QoL total score improved more in OSA patient at 5-year follow-up.

**Conclusions:**

LRYGB is an effective treatment of OSA in obese patients and the achieved beneficial outcomes are maintained at 5-year follow-up.

**Graphical Abstract:**

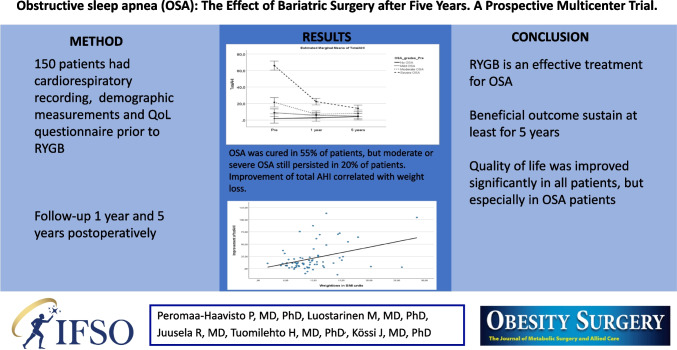

## Introduction

Obstructive sleep apnea (OSA) is one of the most common sleep disturbances. In OSA, upper airway of patient collapses causing a reduction (hypopnea) or cessation (apnea) of breathing during sleep. This leads to sleep fragmentation and intermittent hypoxia during sleep. OSA patients may suffer from daytime sleepiness, slow reaction time, poor concentration and memory, and in long run significantly increased risk for cardiovascular morbidity and mortality. However, in many cases, OSA patient may be completely asymptomatic. The prevalence of OSA in adult population is estimated to be as high as 15 to 20%. In many studies, the prevalence of OSA has been significantly higher (64–97%) especially in bariatric surgery candidates, which are understandable, obesity being the greatest risk factor for OSA. There is also a strong male dominance among OSA patients [1–8].

OSA is highly underdiagnosed disease. At baseline, two out of every three OSA patients were undiagnosed prior to the bariatric surgery [9]. The reason for this may be a lack of symptoms in many OSA patients and a difficulty in screening the disease with questionnaires. OSA is also a slowly progressive disease, and often patients adapt themselves to the current situation. The golden standard for diagnosis of OSA is overnight cardiorespiratory sleep recording demonstrating at least five apnea or hypopnea episodes per hour.

The current first-line treatment for OSA is continuous positive airway pressure (CPAP). It effectively relieves symptoms of OSA but does not cure it. By knowing the key role of obesity in OSA and the fact that approximately 70% of patients with OSA suffer from obesity, the causative treatment should be the treatment of obesity itself. A weight reduction of five percent or more has been shown to cure 61% of patients with mild OSA [10]. However, long-term results of conservative treatment of obesity are often poor, particularly in more severe stages of the disease. Metabolic surgery has proven to affect the resolution of OSA with many different mechanisms. Reduction in adipose tissue in the neck and thorax area relieves the physical pressure of upper airways, reduction in systemic inflammation and insulin resistance has systemic effects, and after certain surgery types (e.g., LRYGB), the alteration of bile and nutrient flow and gut hormone modulation has metabolic effects [11].

The short-term relieving effect of bariatric surgery on OSA has been shown previously [6, 12–15]. However, there is a definite demand for studies focusing on the long-term effect of gastric bypass surgery on OSA. The aim of this prospective, multicenter study was to investigate the effect of LRYGB on OSA 5 years after surgery. In addition, the subjective long-term effect of surgery on Qol was evaluated.

## Patients and Methods

The detailed design of the study was previously reported [9, 15]. Briefly, this study was a prospective multicenter study. The patients were recruited between November 2010 and September 2013. One hundred three patients from Vaasa Central Hospital, 47 patients from Päijät-Häme Central Hospital, and 46 patients from Kuopio University Hospital. However, 5-year data was not available for the patients of Kuopio University Hospital, and they were excluded from the results of this study. Inclusion criteria for the study were the same as for bariatric surgery: age 18–65, BMI more than 35 with co-morbidity, or BMI more than 40. At baseline, both patients with and without OSA were recruited in order to enable comparative analyses between these groups. Exclusion criteria were alcohol or drug abuse, severe eating disorder, severe psychiatric disorder, or other severe disease contra-indicating surgery. Standard overnight cardiorespiratory recording by Embletta® (Embla, Broomfield, CO, USA) was conducted for all the participants at their homes in accordance with accepted guidelines for diagnosing OSA prior to the LRYGB operation. Patients filled out the symptom questionnaires for OSA patients SOS (Snore Outcomes Survey), BNSQ (Basic Nordic Sleep Questionnaire), and ESS (Epworth Sleepiness Scale) and 15D quality of life questionnaires. Written informed consent from the patients was obtained.

The study was approved by the Northern Savo ethics committee and by ethics committees of two other participating hospitals (Vaasa Central Hospital and Päijät-Häme Central Hospital).

### Statistical Analysis

IBM SPSS Statistics 27 was used to carry out statistical analyses. The comparison between different sleep apnea groups was analyzed with independent samples *t*-test and Pearson’s chi-square test. The paired samples *t*-test was used to analyze the statistical significance of changes within the groups, except in those variables where deviation in distribution of values was detected and Wilcoxon test was used instead. In variables with three measuring time points, repeated measures method (general linear model) was used. *p*-value less than 0.05 was considered statistically significant. The change in the prevalence of OSA was analyzed from the data of all patients with a baseline cardiorespiratory recording (*n* = 150), while the change in severity of OSA was analyzed from the data of patients with OSA at the baseline recording (*n* = 111).

## Results

**Fig. 1:**
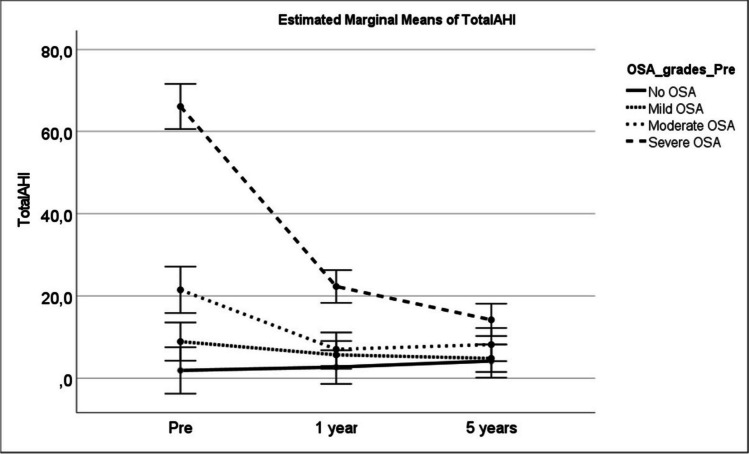
Estimated marginal means of total AHI preoperatively, 1 year and 5 years after operation in different groups stratified according to preoperative OSA status, *p* < 0.001 pre vs. 1 year and pre vs. 5 years, *p* = ns 1 year vs. 5 years (GLM repeated measures). Difference between no OSA and mild OSA *p* = ns., mild OSA vs. moderate OSA *p* = 0.027, all other between groups differences *p* < 0.001 (Tukey’s HSD post hoc test). Number of evaluated patients is 150 at baseline, 133 at 1 year, and 91 at 5 year. Difference between OSA groups were tested by Tukeys post hoc test

**Fig. 2:**
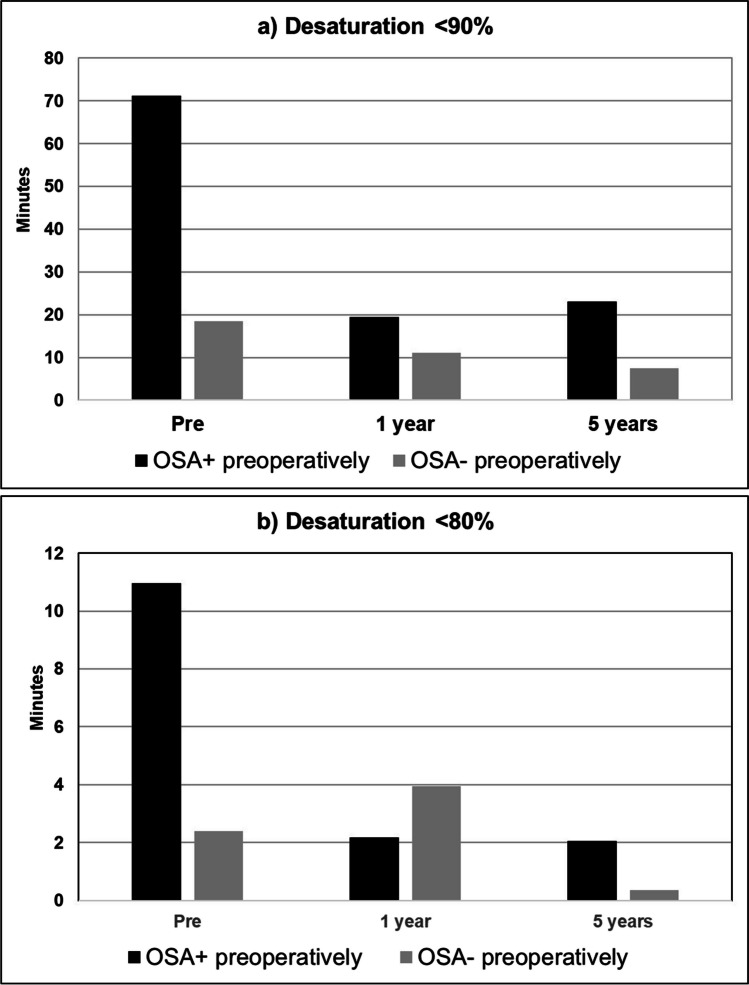
Oxygen desaturation in minutes **a** < 90% and **b** < 80% preoperatively, at 1 year and at 5 years postoperatively in Embletta® monitoring. **a** In patients without OSA preoperatively, all differences were not significant. In patients with OSA preoperatively, *p* was < 0.001 for differences between preoperative and 1 year or 5 years desaturations and ns. for 1 year vs. 5 years. **b** In patients without OSA preoperatively, all differences were not significant. In patients with OSA preoperatively, *p* was < 0,05 for differences between preoperative and 1 year or 5 years desaturations and ns. for 1 year vs. 5 years. Number of evaluated patients is 150 at baseline, 133 at 1 year, and 91 at 5 years

The total of 150 patients were included in this study, 90 (60%) were women and 60 (40%) were men. The diagnostic criteria for OSA were fulfilled by 111 patients (73.3%). When evaluated separately, 91.7% of males and 62.5% of females had OSA diagnosis. The 1-year cardiorespiratory recording was available for 102 of these patients with OSA and the 5-year cardiorespiratory recording for 70 patients. Demographic and anthropometric characteristics of all patients and patients with OSA are shown in Table 1. There was a significant difference (*p* < 0.001) between all measures (weight, BMI, waist, and neck circumference) at 1 and 5 year in all patients and patients with OSA compared to baseline. Cardiorespiratory recording data at baseline, 1-, and 5-year follow-up visits for OSA patients is shown in Table 2. There was a significant difference between all measures at 1- and 5-year follow-up compared to baseline. The mean total AHI decreased from 29.0 at baseline to 8.8 at 5-year visit. In Fig. 1, the change in marginal means of total AHI stratified by OSA is shown preoperatively, at 1-year and 5-year follow-up. The change in desaturation time below 90% and below 80% preoperatively and at 5-year visit is shown in Fig. 2.

**Table 1:** Demographic and anthropometric characteristics at baseline, 1 year, and 5 years after bariatric operation

	Baseline	One year	Five years	*p*-value
Age(years), mean (SD)				
All patients	47.5 (9.7)			
Patients with OSA	49.5 (9.0)			
Weight (kg), mean (SD)				
All patients	127.5 (23.4)	93.6 (18.7)	97.3 (19.8)	< 0.001
Patients with OSA	130.4 (25.2)	96.8 (19.5	101.4 (19.9)	< 0.001
BMI (kg/m^2^), mean (SD)				
All patients	43.9 (5.9)	32.2 (5.0)	33.5 (5.5)	< 0.001
Patients with OSA	44.5 (6.4)	33.0 (5.1)	34.7 (5.4)	< 0.001
Waist circumference (cm), mean (SD)				
All patients	130.1 (14.6)	104.0 (17.7)	107.8 (16.2)	< 0.001
Patients with OSA	133.2 (14.8)	106.9 (18.8)	112.2 (15.8)	< 0.001
Neck circumference (cm), mean (SD)				
All patients	43.9 (4.6)	39.4 (8.6)	39.6 (4.4)	< 0.001
Patients with OSA	45.6 (6.9)	40.6 (9.6)	40.9 (4.3)	< 0.001

All patients and patients with diagnosed OSA at baseline. Number of evaluated patients is 150 at baseline, 133 at 1 year, and 91 at 5 years and corresponding figures for patients with OSA are 111, 102, and 70

**Table 2:** Sleep registration data at baseline, 1 year, and 5 years after bariatric operation of the patients with OSA at baseline

	Baseline (*n* = 111)	One year (*n* = 102)	Five years (*n* = 70)	*p*-value
AHI, total, mean (SD)	29.0 (26.3)	9.7 (10.8)	8.8 (9.9)	< 0.001
AHI supine, mean (SD)	31.8 (28.6)	16.0 (18.4)	13.3 (16.0)	< 0.001
AHI other than supine, mean (SD)	27.5 (30.2)	6.1 (9.4)	6.6 (9.1)	< 0.001
SpO_2_ below 90% (min), mean (SD)	74.6 (93.3)	18.5 (38.2)	20.3 (35.0)	< 0.001
SpO_2_ below 80% (min), mean (SD)	7.6 (24.1)	0.5 (2.8)	0.6 (2.2)	0.01
Heart rate (beats/min), mean, (SD)	67.1 (9.0)	59.8 (8.1)	61.0 (7.1)	< 0.001

Number of evaluated patients are 111 at baseline, 102 at 1 year, and 70 at 5 year

**Fig. 3:**
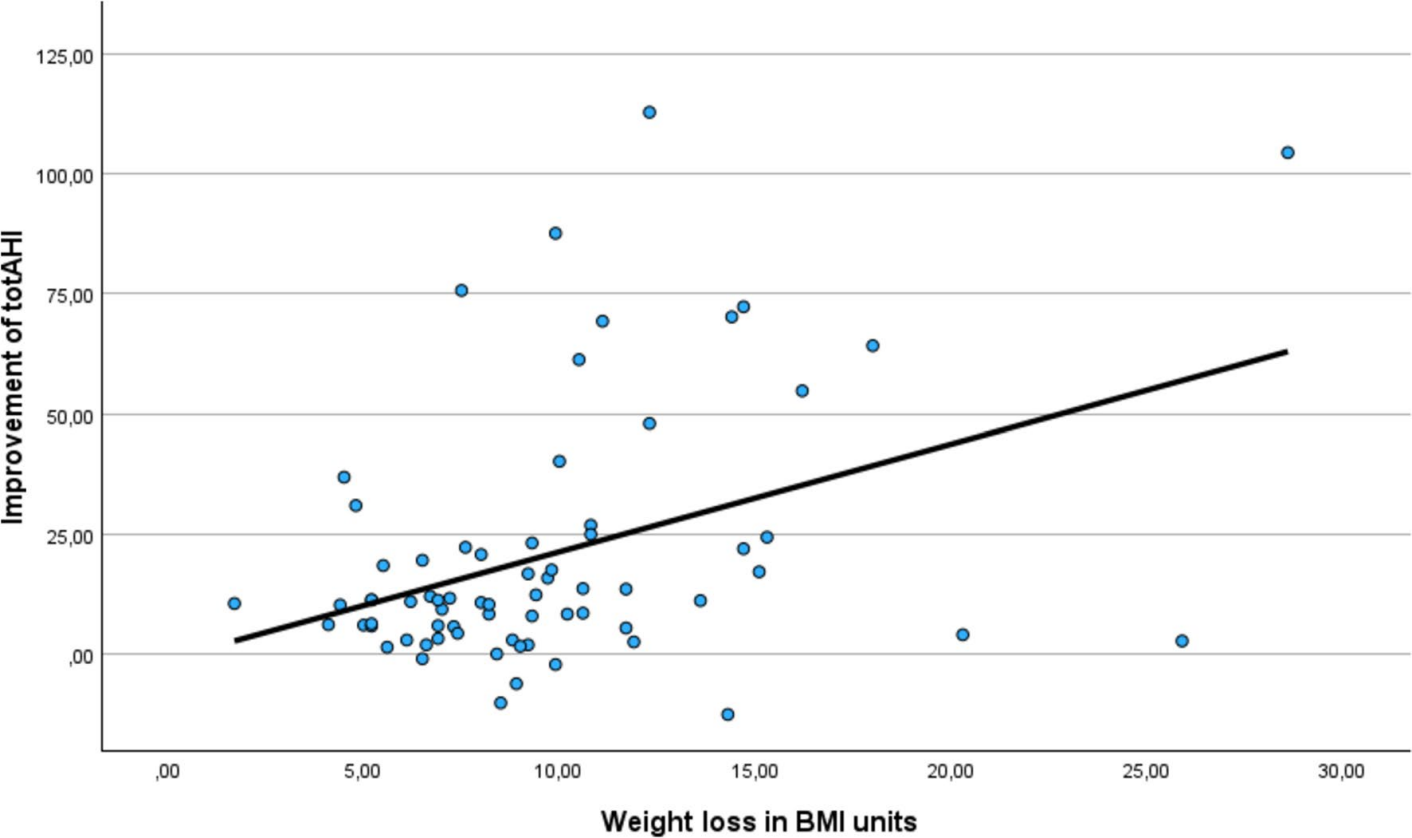
In OSA patients, improvement of total AHI correlated significantly with weight loss. (Pearson correlation 0.395, *p* < 0.001)

The mean weight of patients with OSA in the beginning of the study was 130.4 kg. The mean percent of total weight loss (%TWL) was 25.8% after 1 year and 22.2% after 5 years. The initial mean BMI of the patients with OSA was 44.5 kg/m^2^ decreasing by 11.5 kg/m^2^ over the 1-year and 9.8 kg/m^2^ over 5-year follow-up period. The significant correlation between improvement of total AHI and weight loss is shown in Fig. 3.

The specific distribution of OSA patients due to a severity of OSA at baseline and at 5-year follow-up visit is shown in Table 3. The OSA was cured (AHI < 5) for 38/70 (55.1%) of patients over the 5-year follow-up period. Of these 38 patients with OSA in remission, 23 (60.5%) have had mild, 9 (23.7%) moderate, and 6 (15.8%) severe OSA at baseline. Moderate or severe OSA still persisted in 14 (20%) patients.

**Table 3:** Number of OSA patients in different OSA groups at baseline and 5 years after the bariatric operation

	5 years after bariatric surgery			
Baseline	No OSA	Mild	Moderate	Severe
Severe	6	7	5	3
Moderate	9	8	3	0
Mild	23	3	3	0
Total	38	18	11	3

Patients with sleep registration at 5 years included (*n* = 70). Pearson chi-square test *p*-value 0.01. Number of evaluated patients is 70 at 5 years

Four patients were diagnosed with de novo mild OSA at 1-year visit. Two of them still had mild OSA at 5-year follow-up, but one progressed to moderate and one to severe OSA. All these patients had experienced a significant weight loss, the initial mean BMI was 43.3 kg/m^2^, 31.0 kg/m^2^ at 1-year and 32.4 kg/m^2^ at 5-year visit. Total AHI was 2.45 events/h in the beginning, 9.4 events/h at 1-year and 17.48 events/h at 5-year follow-up visit.

**Fig. 4:**
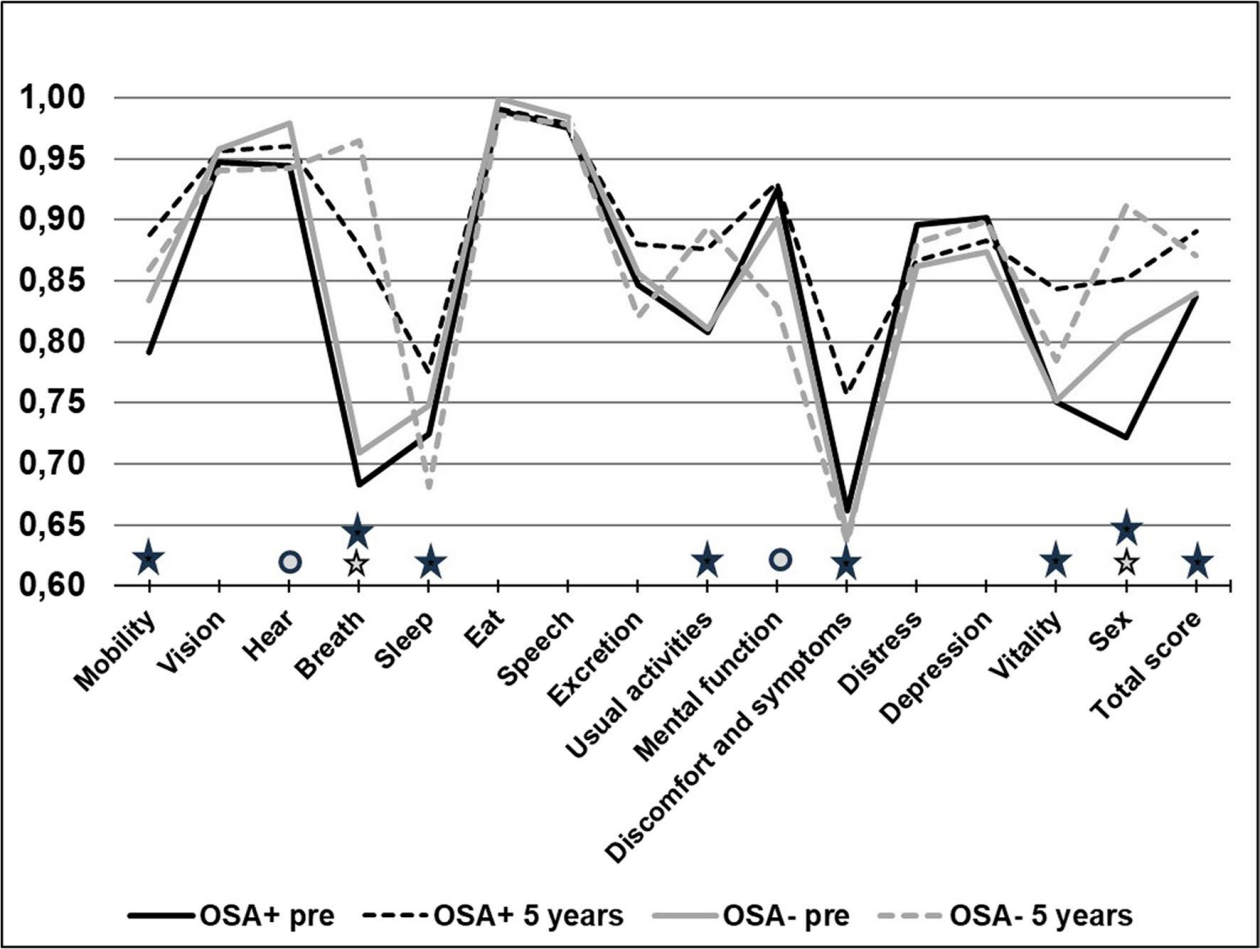
15D quality of life instrument preoperatively and 5 years postoperatively in patients with preoperative obstructive sleep apnea (= OSA +) or without (= OSA-). Change more than 0.035 is regarded as clinically significant. Black star: Statistically and clinically significant improvement preoperative vs. 5 years in OSA + patients. Grey star: Statistically and clinically significant improvement preoperative vs. 5 years in OSA- patients. Grey circle: Statistically and clinically significant deterioration preoperative vs. 5 years in OSA- patients. Number of evaluated patients is 150 at baseline, 133 at 1 year, and 91 at 5 years

A generic 15-dimensional (15D) questionnaire was used to measure health-related quality of life at baseline, 1-year, and 5-year follow-up visits. The 5-year results are shown in Fig. 4. In patients with OSA, a clinically and statistically significant improvement was seen already after 1 year and sustaining over 5-year follow-up in mobility, breathing, sleeping, usual activities, discomfort and symptoms, vitality, and sexual activity in patients. Also the total 15D score improved clinically and statistically in these patients. In patients who did not have OSA, the changes were slightly lesser: two dimensions, breathing, and sexual activity were clinically and statistically improved at 5-year visit, but two dimensions, hearing, and mental function were deteriorated. The total 15D score also improved clinically significantly in patients without OSA.

## Discussion

To our knowledge, this is the first study with reasonable number of patients evaluating the long-term effect of gastric bypass surgery on OSA. Our study demonstrates that bariatric surgery is an effective treatment of OSA even 5 years after the surgery. Three of five patients were considered to have been cured (i.e., they had an AHI < 5), and one out of every four (26.1%) patients were observed to have only mild OSA postoperatively. Furthermore, marked subjective improvements in the QoL were demonstrated.

According to a recent meta-analysis, the short-term effect of bariatric surgery on OSA was associated with an overall rate of remission of 65% [16]. In our study, the corresponding rate was 55% which can be regarded being in line with earlier studies taken into account the long follow-up time of the present study. There are number of studies evaluating the effect of bariatric surgery on OSA, but in most of them, the follow-up time is short, and the number of patients included is small [11–15]. Previous long-term studies including 1 to 36 patients have reported 66–100% improvement of OSA after sleeve gastrectomy [17–20]. Probably, the most comparable study with 65 patients comparing sleeve gastrectomy and LRGYP reported 42% and 45%, respectively, improvement of OSA 10 years after surgery [21]. Our study is well in line with these previous reports.

Not all patients with OSA were cured from the disease, but considering the rate of patients with mild OSA at 5-year follow-up, we can assume that roughly 80% of patients benefit from bariatric surgery. This is essential, since it is well-documented that the severity of OSA is associated with an increased risk for cardiovascular morbidity and mortality [22, 23]. OSA is a chronic disease with a tendency to worsen with time. Based on current knowledge about the evolution of OSA, we believe that weight gain represents a high risk for future progression towards more severe disease. This study provides long-term evidence that sustained weight reduction by LRYGB can not only significantly improve OSA in obese patients but also prevent the progression of OSA, thus reducing the risk for cardiovascular morbidity and mortality.

Since in our study there was a fifth of OSA patients still suffering moderate or severe symptoms of OSA after surgery, it is important to have a postoperative assessment to identify these patients. A postoperative cardiorespiratory recording is recommended for all OSA patients, and it is important to counsel the patients not to discontinue the treatment, e.g., with CPAP only based on symptoms.

To support our belief of reduced risk of cardiovascular morbidity, the present study also showed marked improvements in desaturation times under 90% and 80% and in mean heart rate. In a recent study, the severity of OSA as measured by degree of nocturnal oxygen desaturation is associated with cardiovascular consequences especially in women, who may be more susceptible to the impact of nocturnal hypoxemia [24]. Previous studies have also demonstrated the importance of desaturation levels on OSA-related symptoms and consequences. The severity of sleep-related desaturations has been shown to be more significant contributor to daytime sleepiness compared to AHI [25]. Moreover, night-time desaturation has also been shown to be associated with memory impairment in adults and increased incidence of diabetes [26, 27]. It could be assumed that surgery induced marked decrease in desaturation time is one reason for beneficial overall effects on obesity-related comorbidities.

Obesity and OSA are associated with decreased QoL, and bariatric surgery has been shown to increase it both short- and long-term regardless of indications for surgery [28–32]. Studies comparing the effect of bariatric surgery on QoL in patients with OSA and without OSA are still scarce. In a retrospective study by de Raaff et al. [33], patients with OSA had lower postoperative scores on public distress and work after laparoscopic gastric bypass compared to patients without OSA. This finding is in contrast with our results since we noticed distinct improvements in four dimensions and total score in patients with OSA. All physiological parameters measured in our study, such as AHI, weight, heart rate etc., improved supporting beneficial changes in subjective well-being. Besides the study design, another explanatory factor compared to the earlier study may be the difference in follow-up time. In our study, the follow-up time was longer (5 years vs. 15 months). On the other hand, it has been reported that QoL after surgery improves in the short term but declines slightly after 2 years [30].

Some limitations of the present study have to be addressed. Only 60.7% ( 91/150) of the initial 150 OSA patients at baseline had polysomnography at the 5-year follow-up visit which weakens the statistical power of the study. In OSA patients, the number was a little higher 63.1% (70/111). However, in Finland, bariatric surgery is centralized in larger hospitals, and patients coming to bariatric surgery may live hundreds of kilometers away from the hospital providing a challenge for additional visits. Instead of using in-laboratory polysomnography, portable recording devices (Embletta®) were used in the present study. However, clinical guidelines for the use of portable monitors have been introduced, and there is now a recommendation that portable monitoring may be used as an alternative to polysomnography for the diagnosis and treatment follow-up of OSA [34]. In addition, being a multicenter study, we aimed to standardize all methods used and choose them accordingly. Although these data are encouraging, they need to be replicated in a larger study.

## Conclusions

The LRYGB is an effective treatment for OSA and beneficial outcome sustain at least for 5 years. The postoperative cardiorespiratory recording is still recommended to identify 20% of patients with moderate to severe OSA after 5 years.
